# Familiarity modulates both intra- and interspecific yawn contagion in red-capped mangabeys

**DOI:** 10.1038/s41598-022-15395-0

**Published:** 2022-07-01

**Authors:** Luca Pedruzzi, Juliette Aychet, Lise Le Vern, Veronica Maglieri, Arnaud Rossard, Alban Lemasson, Elisabetta Palagi

**Affiliations:** 1 Department of Biology, University of Pisa, Via A. Volta 6, 56126 Pisa, Italy; 2 Univ Rennes, Normandie Univ, CNRS, EthoS (Éthologie animale et humaine)-UMR 6552, 35000 Rennes, France; 3 Institut Universitaire de France, 75231 Paris, France; 4 Natural History Museum, University of Pisa, Via Roma 79, 56011 Calci, Pisa Italy

**Keywords:** Neuroscience, Psychology

## Abstract

Yawn contagion (YC) is, compared to spontaneous yawning, an evolutionary recent phenomenon probably linked to behavioral synchronization in highly social species that is more likely when it involves familiar subjects. Here, we investigate for the first time in monkeys which factors modulate intra- and interspecific YC. Through an experimental approach, we exposed 17 red-capped mangabeys to video stimuli (Yawn vs Control) depicting familiar/unfamiliar red-capped mangabeys and humans, and unfamiliar hamadryas. We found that mangabeys yawned more often in response to Yawn than Control videos independently from the species depicted, demonstrating both intra- and interspecific YC in the tested species. Moreover, both mangabey and human familiar yawning stimuli evoked a stronger yawning response in the subjects compared to the unfamiliar counterparts. Neither the amount of time spent looking frontally at the screen (probability of stimulus perception) nor the levels of self-directed behaviors (a proxy of anxiety) accounted for the results. In conclusion, we provide the first evidence that in non-human primate familiarity modulates both intra- and inter-specific YC. Stimuli emitted by familiar faces somehow ease the mechanisms underlying YC, and this modulation can also apply to heterospecific subjects when previous shared experiences provide the prerequisites for the development of social bonds.

## Introduction

Spontaneous yawning is an evolutionary ancient trait that is consistent in its presence and expression across several groups of vertebrates^1,2^. Yawning is a multi-functional phenomenon whose functions are probably context-dependent and linked to both physiological as well as behavioral aspects of life^3^. Yawn contagion (YC), the ability to respond with a yawn to others’ yawns, is instead an evolutionary more recent phenomenon^4,5^. Recent findings suggest that YC can have an adaptive social role in bodily coordination, mood alignment and alertness increase^6,7^. Both experimental and observational evidence indicate sociality and cooperation as triggering factors for YC to evolve in different taxa^6,8–15^. YC can also be present between subjects belonging to different species (i.e., interspecific YC^16–18^) thus indicating a certain level of flexibility of its underlying mechanisms.

Familiarity shared between the interacting subjects seems to play a role in the modulation of stimuli contagiousness, and an animated debate exists on the proximate causes at the basis of such modulation (emotional closeness vs attentional bias,^19,20^). Relevant clues supporting the linkage between yawn and emotional contagion (a building-block of empathy,^21^) come from psychological studies. People showing higher susceptibility to contagious yawning do better at making inferences about mental states^22^ and exhibit fewer schizotypal traits^23^. Moreover, when exposed to yawn vs control stimuli, higher activity in empathy-related neuronal areas was recorded in people scoring highly on empathy (^24^ but note that the authors did not measure the contagion itself^25^). The emotional bias hypothesis predicts that the degree of emotional involvement with a first yawner reflects the observer’s susceptibility to respond with a yawn^4,21^. Instead, the attentional bias hypothesis predicts that YC is merely linked to the higher levels of social attention that observers devote to more relevant subjects (i.e., familiar or dominant)^25^. More recently, Gallup et al.^26^ found that those people scoring high levels of psychopathic traits, which are associated with reduced affective empathy, also showed low levels of YC, with the yawn response not depending on the attentional level. Although this dualistic conceptual approach is present in literature, the two aspects are difficult to disentangle since probably both attentional and affective processes play a role in the modulation of YC^20^. Whatever the proximate factors at the basis of YC modulation, several studies show how affiliation and kinship increase the susceptibility to respond to others’ yawns in several species^10–12,14,16,18,27^. Concurrently, the available data do not always go in the same direction, with different examples of social closeness not influencing YC^28–31^, thus challenging the view about the positive effect of familiarity on the phenomenon. Species scoring low affiliation and/or high degrees of ingroup competition do not seem to show YC^32,33^ and, moreover, while xenophilic species show similar levels of YC towards known and unknown individuals^34^, species classified as xenophobic show contagion strictly towards ingroup subjects^18^. Another factor that has been found to influence the YC distribution is the sex of the interacting subjects, especially in those species where bonding is sex-biased (e.g., bonobos, 10; wolves^12^). Specifically, wolf females showed a faster yawning response when sharing a strong bond with the first yawner, such difference was not found in males^12^.

Here, we aim at testing the effect of familiarity on both intra- and interspecific YC in monkeys due to the absence of data covering these intermingled aspects in non-ape species. Filling the gap is necessary if we want to properly understand the evolutionary aspects of the phenomenon. Our model is the red-capped mangabey (*Cercocebus torquatus*), a highly sexually dimorphic species living in multi-male multi-female groups variable in size and characterized by fission–fusion dynamics^35^. Although clear hierarchies exist in social groups, the dominance relationships are generally relaxed thus predicting a certain level of tolerance^36^. *C. torquatus* further possesses a rich repertoire of visual signals with a large variety of yawning types^37^. The peculiarity of their social life and the variability of yawn performance make the species a valuable candidate to test hypotheses on the proximate factors leading to YC. To accomplish the goal, we showed red-capped mangabeys video stimuli from familiar and unfamiliar individuals belonging to three species (*Cercocebus torquatus*, *Papio hamadryas*, *Homo sapiens*). In our protocol, we also took into consideration the probability of stimulus perception (i.e., how long the subjects were frontally looking at the screen) as well as the anxiety level of the subjects when exposed to the different stimuli (i.e., self-directed behaviours^38^), as both factors can act as confounding factors for YC and its modulation^25,39^.

If YC is sensitive to the phylogenetic closeness between the interacting subjects (hypothesis 1), we expect red-capped mangabeys to be more susceptible to yawns produced by conspecifics and hamadryas baboons than to yawns produced by humans (prediction 1).

If YC is modulated by previous experiences (i.e., familiarity) between the interacting subjects (hypothesis 2), we expect red-capped mangabeys to be more susceptible to yawns produced by familiar red-capped mangabeys and humans than to those produced by the unfamiliar counterparts (prediction 2).

## Methods

### Experimental procedures

17 adult captive-born red-capped mangabeys (10 males, 7 females) hosted at the Station Biologique de Paimpont (University of Rennes 1) were involved in the study. The animals occupy enclosures with indoor and outdoor spaces and live in groups with different compositions ranging from one-male to all-male groups. The experimental sessions took place in the indoor enclosure (where the temperature is kept at 22 °C), while non-tested subjects remained outside. The animals, well-habituated to be separated, were tested only when they spontaneously entered the test area (see “Supplementary Information” for further details).

The video stimuli showed three different species (red-capped mangabeys, humans, and hamadryas baboons). We generated both Yawn and Control videos depicting unfamiliar hamadryas and unfamiliar/familiar mangabeys and humans, for a total of 10 video stimuli per tested subject (i.e., unfamiliar and familiar mangabeys Control/Yawn, unfamiliar and familiar humans Control/Yawn, hamadryas Control/Yawn). Indeed, hamadryas baboons are unknown to our tested subjects and to the species in general (no geographical range overlap), thus only unfamiliar stimuli depicting baboons were prepared. The videos were mute 5-min videos, composed by 2 alternating videos depicting 2 different individuals. The two videos alternated each other along the whole duration of the stimulus (i.e., 5 min) and were separated each time by a black blank screen (random duration, 1–3 s). Each 5-min video included 55 yawning events. Mangabey videos were collected at the Station Biologique de Paimpont (Paimpont, France) and hamadryas videos were recorded at the Hellabrunn Zoo (Munich, Germany). To prepare the human stimuli, videos were recorded ad hoc from nine volunteers. To assess the spontaneity level of human yawns, we asked seven other volunteers to score each yawn (1 = fake yawn; 10 = real yawn). Only 65 out of the 85 yawns scoring higher than 5 were used. Since hamadryas were completely unfamiliar to the tested subjects only for mangabeys and humans we designed video stimuli depicting familiar or unfamiliar individuals ad hoc for each subject. The mangabeys in the video were considered as familiar if they have lived together or in adjacent cages with the tested subject within the last 5 years. Human familiar subjects were the caretakers who have been daily interacting with animals for more than 1 year^18^. The Yawn videos included male yawns from different age classes, both adults and juveniles, emitted with different mouth opening degrees, face orientations and backgrounds. The choice of male yawns for the three species derives from the fact that it was not possible to have unfamiliar mangabey females for each of the subjects (specifically, all the females lived in the same enclosure for at least a period within the last 5 years). Then, to make the stimuli from the different species comparable, we also included male yawns for humans and hamadryas. All the yawns were performed under relaxed conditions (e.g., excluding feeding times, in the absence of aggression preceding the yawn stimulus). For all the three species used as stimuli, each Control video stimulus included two alternated videos of the same 2 individuals depicted in the Yawn video stimulus. The videos used for the two conditions (Yawn/Control) were extracted from the same original video. Hence, in both of them the individuals had identical body orientation, context and background, with the only difference being that in the Control videos they chewed or opened/closed their mouth without yawning. The video stimuli depicting the three different species were prepared to be as much standardized as possible. Mangabeys and hamadryas were recorded in their different enclosures (outdoor and indoor areas). Human videos were thus recorded in variable areas, with variable lighting, head orientation and proportion of the body filmed, so as to reproduce the conditions of the other two species and to make the stimuli comparable in terms of quality of depicted yawns. We prepared the video stimuli ad hoc for each tested subject depending on the research question (i.e., species, degree of familiarity). The two filmed individuals were randomly extracted from recorded videos including 22 red-capped mangabeys, 9 humans, 10 hamadryas baboons.

We introduced a two-step habituation phase before the experimental sessions to i) reduce neophobia towards the equipment (5-h presence of the screen kept off in the indoor enclosure) and ii) make animals more comfortable in watching images in the screen (5-h presence to the screen showing neutral images of the environment surrounding the animals’ enclosures—with no humans or animals depicted). Each experimental session lasted 10 min: 2-min blank screen (pre-stimulus), 5-min of video stimulus, and 3-min blank screen (post-stimulus, to account for latency in the response^40^). The 10 stimuli (i.e., unfamiliar and familiar mangabeys Control/Yawn, unfamiliar and familiar humans Control/Yawn, hamadryas Control/Yawn) were randomly presented in two time slots (i.e., morning and afternoon). Each subject had 5 sessions in the morning and 5 in the afternoon (random division, no more than a session per day per subject). Within each time slot the order in which the 5 stimuli were presented to each subject across different days was also randomized. Since yawns performed by the subjects outside of the enclosure could have been possibly visible to the tested subject, all these yawns and their exact time of occurrence were collected (*Outside Yawns*). Further details on the experimental protocol are provided as “Supplementary Information”.

### Video analysis and statistics

**Figure 1:**
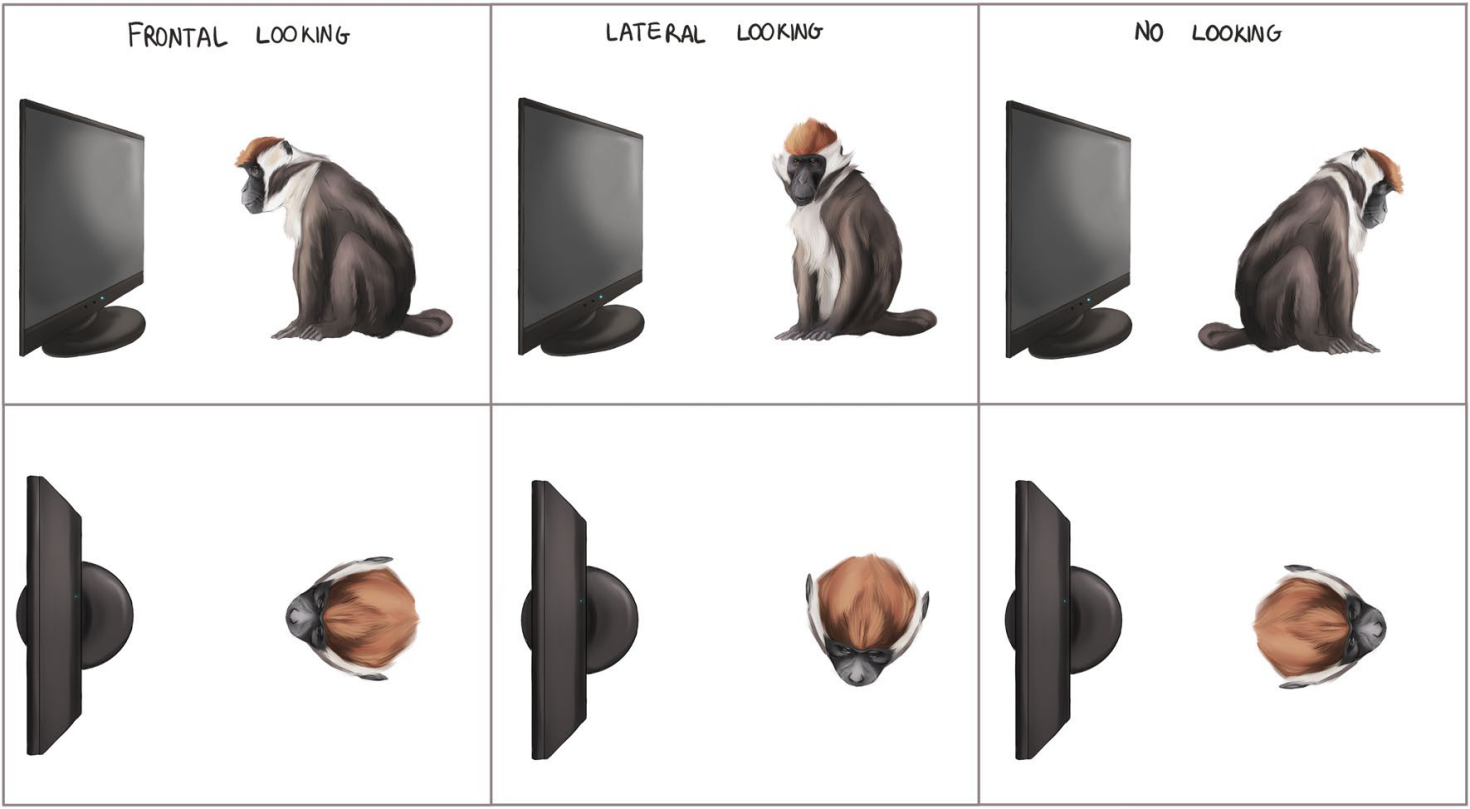
Codification of stimuli perception by means of looking directions during the experimental sessions. Under the FL condition the head of the subject had to be directed towards the screen. Credits Fosca Mastrandea.

When analyzing the videos of the sessions, we coded for the presence/absence of yawns (Yawn Response, YR) and the amount of time the tested subject looked frontally at the screen when showing the video stimulus (Frontal Looking, FL; Fig. 1). The other conditions shown in Fig. 1 and those in between them (in terms of head orientation) were parsimoniously not coded as FL. We also recorded self-directed behaviors (SDB, scratching, self-grooming, head-shaking) during the sessions, considered as good indicators of anxiety in primates^38^. We ran two Linear Mixed Models (glmmTMB 1.2.5042 package on R) to evaluate which variables affect the rates of FL and of SDBs. Then, we ran a Generalized Linear Mixed Model (glmmTMB 1.2.5042) to evaluate which variables affected the YR. According to the hypotheses tested, we split the analysis into two steps. Firstly, we focused on the effect of the species in the video (unfamiliar individuals of the three different species—Model set_1_). Secondly, we focused on the effect of familiarity (video stimuli of unfamiliar and familiar mangabeys and humans—Model set_2_). In all the models the identity of the tested subject (i.e., ID) was included as a random factor. See “Supplementary Information” for further details on statistics used.

### Model set_1_—YC is sensitive to the phylogenetic closeness between unfamiliar subjects (hp 1)

#### Model_1a_—FL (response variable, Gaussian error distribution)

The fixed factors considered were: *Sex* of the tested subject, *Species* in the video (Mangabey/Human/Hamadryas), *Condition* (Yawn/Control), *Time* of the session (Morning/Afternoon), *Order* of the stimuli presentation.

#### Model_1b_—SDB (response variable, Gaussian error distribution)

The fixed factors included were those considered in Model_1a_.

#### Model_1c_—YR (response variable, binomial error distribution)

The fixed factors were the same of Model_1a_ with the addition of *Outside Yawns* and *FL*.

### Model set_2_—YC is modulated by familiarity between subjects (hp 2)

#### Model_2a_—FL (response variable, Gaussian error distribution)

The fixed factors considered were: *Sex* of the tested subject, *Species* in the video (Mangabey/Human), *Condition* (Yawn/Control), *Time* (Morning/Afternoon), *Order* of the stimuli presentation, *Familiarity* of the stimulus (Familiar/Unfamiliar).

#### Model_2b_—SDB (response variable, Gaussian error distribution)

The fixed effects were the same considered in Model_2a_.

#### Model_2c_—YR (response variable, binomial error distribution)

The fixed factors were the same considered in Model_2a_, with the addition of *Outside Yawns* and *FL*.

We first included in the models meaningful interactions between the predictors: Sex*Condition, Sex*Species (Model set_1_) and Sex*Condition, Sex*Species, Familiarity*Species, Familiarity*Sex (Model set_2_). As discussed in the “Supplementary Information”, the interactions were included in each final model only if they were significant.

### Ethics

The present study consisted in a non-invasive protocol conducted with captive mangabeys housed at the Station Biologique de Paimpont (University Rennes 1, France), where animal facilities and animal care procedures are regularly monitored by the responsible local authorities (Housing agreement for research D35-211-18, delivered by the “Direction Départementale de la Cohésion Sociale et de la Protection des Populations” (DDCSPP)). The ethical authorisations for conducting research studies in this facility are given by the CREEA Ethic committee (“Comité Rennais d’Ethique en matière d’expérimentation animale”), which is the legal representative for all animal experimentations from institutions located within this geographical area. This committee is registered with the "Comité National de Réflexion Ethique sur l'Expérimentation Animale" under the number 07. The current research protocol has been approved by the CREEA Ethic committee under the reference APAFIS#24418-2020012319192625 v2. This study is reported in accordance with ARRIVE guidelines. All human volunteers filmed to constitute the video stimuli gave an informed consent to the use of their images for this research project and were informed about their rights concerning the treatment of their personal data, in accordance with the General Data Protection Regulation (RGPD) compliances. The treatment and storage of these personal data were declared and approved by the Data Protection Officer (DPO) of the University of Rennes 1. Human ethic committees equivalent to IRBs exist at national level but no such approval was required for our study design according to the French regulations since we just used video recordings of humans.

## Results

### Model set_1_—YC is sensitive to the phylogenetic closeness between unfamiliar subjects (hp1)

#### Model_1a_

The full model (fixed variables and interactions) significantly differed from the null one (X^2^_9_ = 22.233, P = 0.008, R^2^_marginal_ = 0.231, R^2^_conditional_ = 0.511). Since both the interactions were not significant (P_Sex*Species_ = 0.939; P_Sex*Condition_ = 0.057), we ran the final LMM excluding them. In Model_1a_, the full model significantly differed from the null one (X^2^_6_ = 18.495, P = 0.005; R^2^_marginal_ = 0.214, R^2^_conditional_ = 0.490). The variables *Sex* and *Species* had a significant effect on *FL* (Table 1a). The Tukey test revealed that the subjects looked more at the videos depicting conspecifics than at those showing humans (t-ratio = − 2.847; df = 93; p = 0.015) and baboons (t-ratio = -2.320; df = 93; p = 0.058) (Fig. S1a). Males looked significantly more at the screen than females (mean ± SE: 0.420 ± 0.028 vs 0.254 ± 0.028) (Fig. S1b).

**Table 1:** (a) Estimated parameters (Estimate), Standard Error (SE), and results of the likelihood ratio tests (χ^2^) of the LMM (gaussian distribution) with Frontal Looking as response variable (Model_1a_). (b) Estimated parameters (Estimate), Standard Error (SE), and results of the likelihood ratio tests (χ^2^) of the GLMM (binomial error distribution) with Yawn Response as response variable (Model_1c_). For both the models the sessions were 102. Significant values are in bold.

Fixed factors	Estimate	SE	*df*	*X*^*2*^	*p value*
**(a) Frontal looking. Variance for the random factor (tested individual) = 0.010, SD = 0.102**					
Intercept	0.609	0.058	–	–	–
Species			2	8.825	**0.012**
Species (human)^a,b^	− 0.017	0.035			
Species (mangabey)^a,b^	0.079	0.034			
Condition (yawn)^a,b^	0.049	0.028	1	3.086	0.079
Sex (female)^a,b^	− 0.198	0.075	1	5.692	**0.017**
Time (afternoon)^a,b^	− 0.030	0.029	1	1.056	0.304
Order of the session	− 0.005	0.008	1	0.330	0.566
**(b) Yawn response. Variance for the random factor (tested individual) = 0.518, SD = 0.720**					
Intercept	− 0.040	1.074	–	–	–
Species			2	0.780	0.677
Species (human)^a,b^	− 0.379	0.593			
Species (mangabey)^a,b^	0.123	0.593			
Condition (yawn)^a,b^	1.287	0.507	1	7.182	**0.007**
Sex (female)^a,b^	− 1.683	0.700	1	6.527	**0.011**
Time (afternoon)^a,b^	− 0.857	0.518	1	2.951	0.086
Order of the session	0.207	0.142	1	2.199	0.138
Frontal looking	0.177	1.364	1	0.017	0.897
Number of outside yawns	− 0.056	0.164	1	0.117	0.733

^a^Estimate ± SE refers to the difference of the response between the reported level of this categorical predictor and the reference category of the same predictor.

^b^These predictors were dummy coded, with the “Species (Hamadryas)”, “Condition (Control)”, “Sex (Male)”, “Time (Morning)” being the reference categories.

#### Model_1b_

There was no difference between the full (fixed variables and interactions) and null model (X^2^_9_ = 15.012, P = 0.091). Similarly, no significant difference was found between the final full and null model (X^2^_6_ = 3.484, P = 0.746). During the entire 8-min block of each session the time spent performing *SDB*s scored low values (mean ± SE: 21.181 ± 2.891 s). This allowed us not to include *SDB* in Model_1c_.

#### Model_1c_

**Figure 2:**
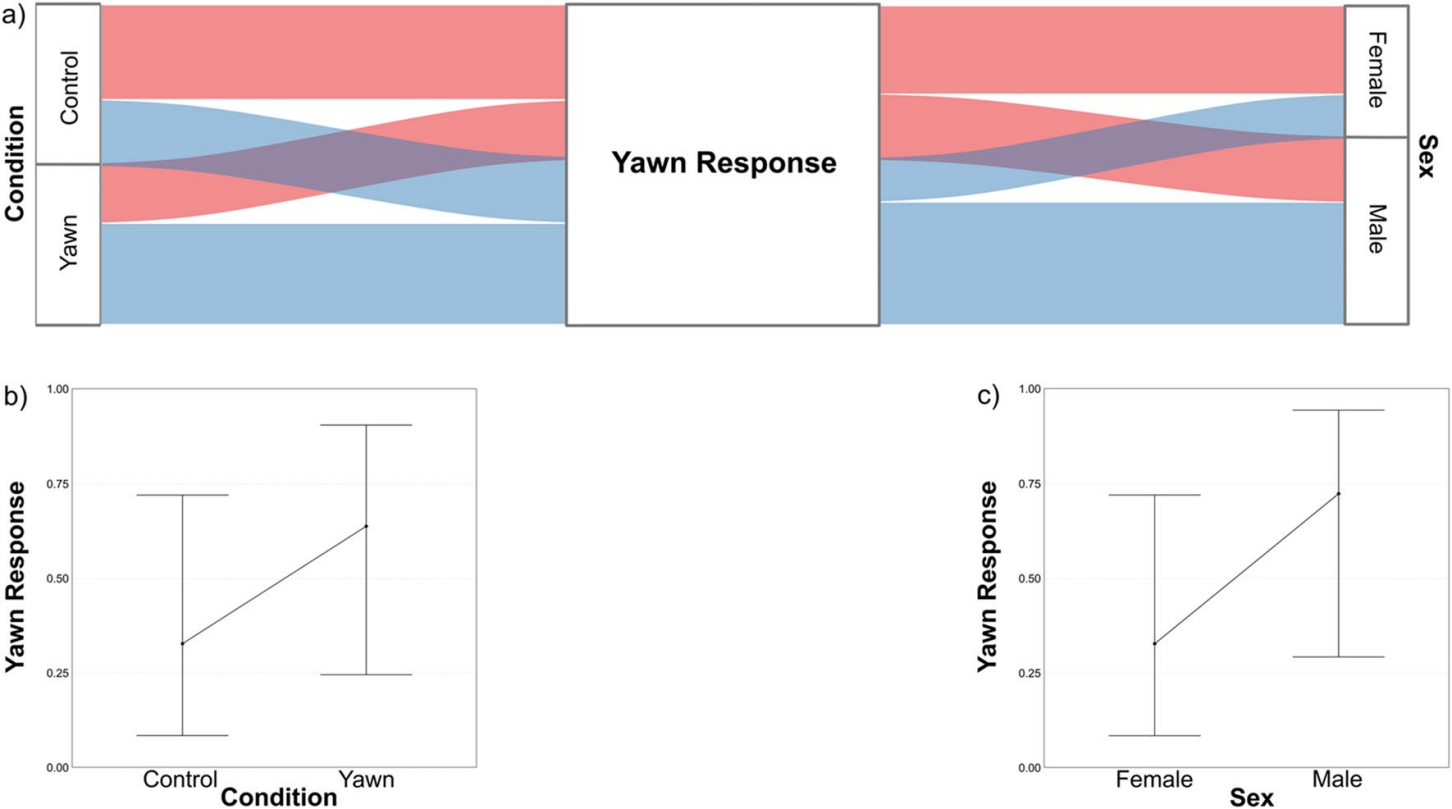
Results of Model_1c_ (Yawn Response as response variable) showing the effect of the significant predictors. (**a**) Alluvial plot showing the occurrence of Yawn Response (Blue streams = presence; Pink streams = absence) for each level of the factors “Condition” (Yawn vs Control) and “Sex” (Male vs Female). (**b**) Effect of the variable Condition on the Yawn response. (**c**) Effect of the variable Sex on the Yawn Response. Bands represent the confidence interval.

The full model (fixed variables and interactions) significantly differed from the null one (X^2^_11_ = 19.731, P = 0.049, R^2^_marginal_ = 0.278, R^2^_conditional_ = 0.377). Since both the interactions were not significant (P_Sex*Species_ = 0.734; P_Sex*Condition_ = 0.392), we ran the final GLMM excluding them. In Model_1c_, the full and the null model significantly differed (X^2^_8_ = 18.435, P = 0.018; R^2^_marginal_ = 0.258, R^2^_conditional_ = 0.359). The *YR* was significantly influenced by the Condition and Sex variables (Table 1b, Fig. 2a). Under the Yawn condition, the likelihood of *YR* was about three times higher (odds ratio = 3.62) compared to Control condition (Fig. 2b). Overall males yawned more than females (Fig. 2c). Neither the *Species* (P = 0.677) nor the rate of *FL* (P = 0.897) affected the likelihood of *YR*.

### Model set_2_—YC is modulated by familiarity between subjects (hp 2)

#### Model_2a_

The full model (fixed variables and interactions) significantly differed from the null one (X^2^_11_ = 45.804, P < 0.0001, R^2^_marginal_ = 0.293, R^2^_conditional_ = 0.571). Since the interactions were not significant (P_Sex*Species_ = 0.686; P_Sex*Condition_ = 0.282, P_Familiarity*Species_ = 0.178; P_Familiarity*Sex_ = 0.574), we ran the final LMM excluding them. In Model_2a_, the full model significantly differed from the null one (X^2^_6_ = 42.566, P < 0.0001; R^2^_marginal_ = 0.283, R^2^_conditional_ = 0.558). *Familiarity*, *Condition*, *Sex*, and *Species* significantly affected *FL* rates (Table 2a). The tested subjects looked more at videos depicting unfamiliar than familiar subjects (mean ± SE: 0.367 ± 0.027 vs 0.246 ± 0.018) (Fig. S2a) and at Yawn compared to Control videos (Fig. S2b). Males looked at the screen for longer than females (Fig. S2c), and conspecifics elicited a higher *FL* than humans (Fig. S2d).

**Table 2:** (a) Estimated parameters (Estimate), Standard Error (SE), and results of the likelihood ratio tests (χ^2^) of the LMM (gaussian distribution) with Frontal Looking as response variable (Model_2a_). (b) Estimated parameters (Estimate), Standard Error (SE), and results of the likelihood ratio tests (χ^2^) of the GLMM (binomial error distribution) with Yawn Response as response variable (Model_2c_). For both the models the sessions were 136. Significant values are in bold.

Fixed factors	Estimate	SE	*df*	*X*^*2*^	*p value*
**(a) Frontal looking. Variance for the random factor (Tested individual) = 0.009, SD = 0.096**					
Intercept	0.550	0.052	–	–	–
Species (mangabey)^a,b^	0.068	0.021	1	10.073	**0.002**
Condition (yawn)^a,b^	0.045	0.021	1	4.518	**0.034**
Sex (female)^a,b^	− 0.140	0.052	1	6.056	**0.014**
Familiarity (familiar)^a,b^	− 0.100	0.021	1	21.012	**0.000**
Time (afternoon)^a,b^	− 0.027	0.022	1	1.523	0.217
Order of the session	− 0.007	0.005	1	2.262	0.133
**(b) Yawn response. Variance for the random factor (Tested individual) = 0.172, SD = 0.415**					
Intercept	− 1.190	0.930	–	–	–
Species (mangabey)^a,b^	0.156	0.455	1	0.118	0.731
Condition (yawn)^a,b^	2.809	0.529	1	41.232	**0.000**
Sex (female)^a,b^	− 1.231	0.579	1	4.863	**0.027**
Familiarity (familiar)^a,b^	0.973	0.481	1	4.260	**0.039**
Time (afternoon)^a,b^	− 0.734	0.455	1	2.680	0.102
Order of the session	0.094	0.096	1	0.971	0.324
Frontal looking	0.212	1.443	1	0.021	0.884
Number of outside yawns	0.045	0.111	1	0.165	0.685

^a^Estimate ± SE refers to the difference of the response between the reported level of this categorical predictor and the reference category of the same predictor.

^b^These predictors were dummy coded, with the “Species (Human)”, “Condition (Control)”, “Sex (Male)”, “Time (Morning)”, “Familiarity (Unfamiliar)” being the reference categories.

#### Model_2b_

There was no difference between the full (fixed variables and interactions) and null model (X^2^_11_ = 14.617, P = 0.201). Similarly, no significant difference was found between the final full and null model (X^2^_7_ = 9.711, P = 0.206). During the 8-min block of each session the time spent performing *SDBs* scored low values (mean ± SE: 18.974 ± 2.130 s). This allowed us not to include *SDB* in Model_2c_.

#### Model_2c_

**Figure 3:**
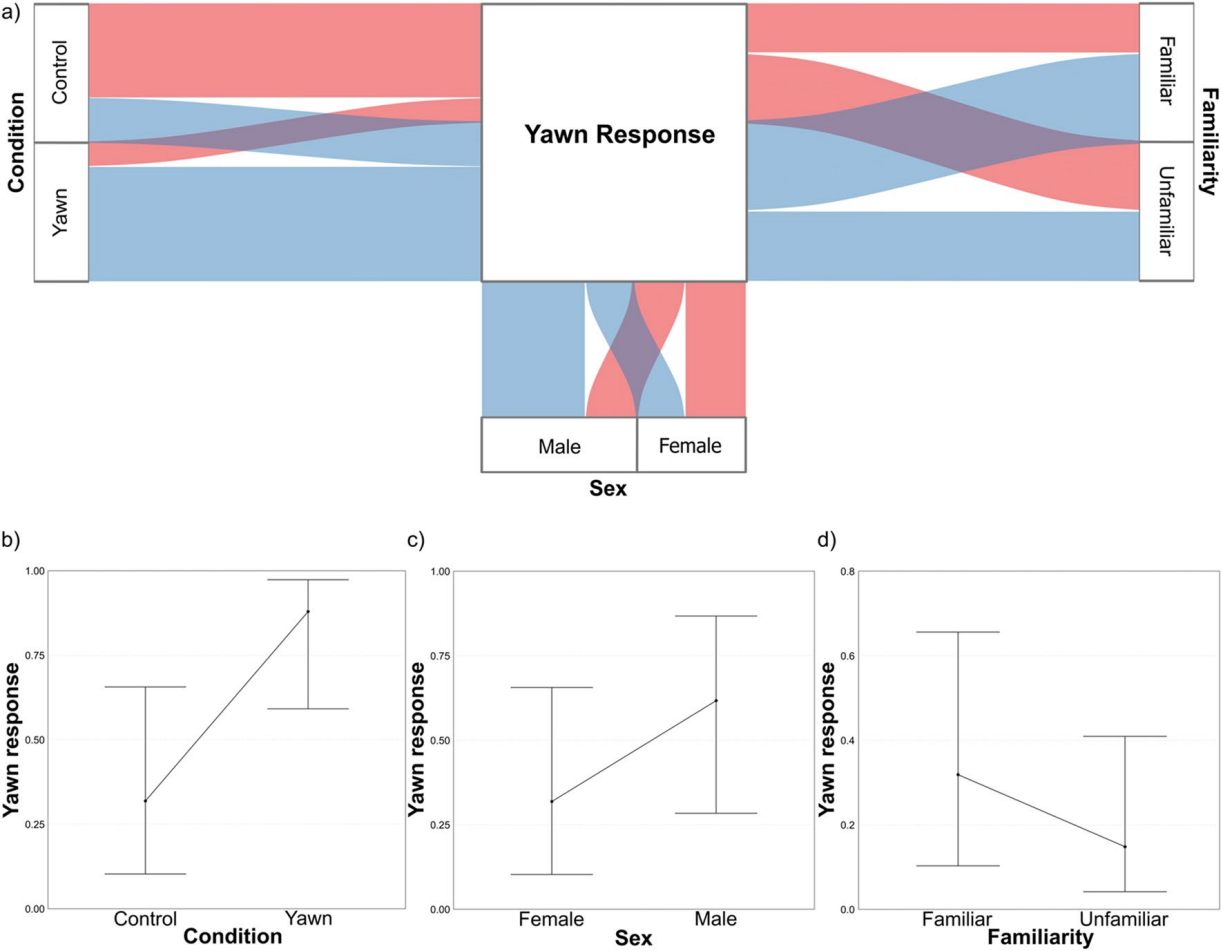
Results of Model_2c_ (Yawn Response as response variable) showing the effect of the significant predictors. (**a**) Alluvial plot showing the occurrence of Yawn Response (Blue streams = presence; Pink streams = absence) for each level of the factors “Condition” (Yawn vs Control), “Sex” (Male vs Female), and “Familiarity” (Familiar vs Unfamiliar). (**b**) Effect of the variable Condition on the Yawn Response; (**c**) Effect of the variable Sex on the Yawn Response; (**d**) Effect of the variable Familiarity on the Yawn Response. Bands represent the confidence interval.

The full model (fixed variables and interactions) significantly differed from the null one (X^2^_11_ = 55.098, P < 0.0001, R^2^_marginal_ = 0.454, R^2^_conditional_ = 0.491). Since all interactions were not significant (P_Sex*Species_ = 0.832; P_Sex*Condition_ = 0.329, P_Familiarity*Species_ = 0.462; P_Familiarity*Sex_ = 0.529), we ran the final GLMM excluding them. In Model_2c_, the full model strongly differed from the null one (X^2^_8_ = 53.516, P < 0.0001; R^2^_marginal_ = 0.444, R^2^_conditional_ = 0.471). *Sex*, *Condition*, and *Familiarity* significantly affected the *YR* (Table 2b; Fig. 3a). The likelihood of *YR* was more than 16 times higher in Yawn than Control condition (odds ratio = 16.60) (Fig. 3b). Overall males yawned more than females (Fig. 3c). The likelihood of yawning was more than twice higher (odds ratio = 2.64) when exposed to familiar rather than unfamiliar subjects (Fig. 3d). All the tested mangabeys emitted at least a yawn in response to familiar subjects’ yawns. Neither *FL* (p = 0.884) nor *Species* (p = 0.731) affected the likelihood of *YR*.

## Discussion

Here, we found that red-capped mangabeys yawned more often to videos depicting yawning individuals than to control videos, thus demonstrating yawn contagion (YC) in this species. Since self-directed behaviors (SDB, a proxy of anxiety) were not affected by neither of the factors considered, this rules out the possibility that yawning response (YR) was a by-product of anxiety. This result contrasts with those from other studies where the perception of yawn stimuli produced an increase of both YR and SDB^39,41^. Our finding also seems to suggest that mangabeys do not perceive yawning behavior as a negative stimulus, differently from what has been found in humans^42^.

The YR was not affected by the amount of frontal looking (FL), thus suggesting that in the species the probability of responding to a yawn cannot be merely explained by the quantitative perception of the stimulus (e.g., for how long you perceive it). Male YR was higher than female YR independently from the species depicted and from the condition of the video stimulus. The higher male tendency to spontaneously yawn is not surprising in those primate species showing remarkable sexual dimorphism in canine size^37,43^. Here, males yawned more often than females both when exposed to Control and Yawn stimuli; importantly, the effect of the Yawn condition in increasing the likelihood of yawning did not differ between the two sexes (i.e., the interaction term Sex*Condition was not significant in the models), with yawn stimuli producing a comparable YC effect in both males and females. Our animals showed similar levels of susceptibility to yawns emitted by conspecific and heterospecific subjects thus demonstrating both intra- and inter-specific YC (prediction 1 not supported). Mangabeys frontally looked for longer the videos depicting conspecific than heterospecific subjects, probably related to the greater socio-ecological relevance for mother-reared primates to strictly monitor members of their own species^42,44,45^. A possible limit of the study is that we could not use eye-tracking techniques, preventing us from actually making conclusions on the attentional state of the subjects. However, we can confidently assume that the longer the animals looked with their head frontally towards the screen, the higher the probability for them actually looking at the individual depicted in the video (which occupied a good portion of the screen and was easily visible). Moreover, since YC does not require an active and conscious attentional and perceptive state of the yawn stimulus^46^, we can affirm that when the mangabey looked frontally at the screen the stimulus was at least passively perceived.

The literature does not provide data on inter-specific YC deriving exclusively in response to unfamiliar subjects/species. Although direct comparisons are difficult, our data are consistent with the at least partial automaticity of motor mimicry phenomena^4,47^, which allows a basal likelihood of contagion independently from the identity and the social features of the trigger subject. Our data can be discussed by two not mutually exclusive interpretations. The consistency of yawning motor patterns (i.e., stereotypy) across different primate species^48^ might explain the similar efficacy in stimulating a yawning response in the receiver. It is also possible that monkeys, as it occurs in humans^8^, might be able to automatically generalize the yawning stimulus whatever its origins.

Our second set of data adds an important piece of information about the phenomenon in mangabeys. When familiarity comes into play, it scores the highest YC rates (prediction 2 supported). Indeed, compared to the unfamiliar counterparts, both familiar mangabey and human yawning stimuli evoked a higher YR in the tested subjects and this held for both males and females. This higher susceptibility towards familiar yawns was not clearly due to a higher probability of familiar stimulus perception, because mangabeys spent more time frontally looking unfamiliar rather than familiar individuals, possibly due to the potential adaptive implications involved when facing novel stimuli^18,49,50^. Similar results have been recently found in great apes, with chimpanzees and gorillas being more attracted by novel than familiar human faces^51^. The familiarity bias in FL that we found is not in contrast with the higher level of FL devoted to conspecific than to heterospecific subjects; indeed, competition for resources is expected to be higher between subjects belonging to the same species (complete ecological niche overlap) but belonging to different social groups, and thus stimuli from unfamiliar conspecifics should be more attentively monitored. During YC, one can share the state of the other based on a perceived motor pattern, through an automatic distributive associative process, which may get easier when the stimuli come from familiar faces^52^. Hence familiarity with the yawner, independently from the species it belongs to, may potentiate the Perception Action Mechanism at the basis of motor resonance phenomena^21^.

In chimpanzees, Campbell and de Waal^18^ reported a familiarity-biased contagiousness only towards conspecifics, but not towards humans, a species to which chimpanzees were well habituated. Conversely, in our study, not only were red-capped mangabeys susceptible to yawns produced by conspecifics and humans, but they also showed a comparable familiarity bias towards both species. Similar hypotheses have been so far rarely tested in primates^18^ and a modulation in the contagiousness of heterospecific yawns was only found in domesticated dogs (16, but see also 28) where, on the other hand, no study investigated intraspecific YC. Our data thus represent the first evidence in mammals of familiar modulation on YC operating at both intra- and interspecific level. Unfamiliar humans seem to be perceived by mangabeys as something equivalent to the ‘outgroup’ conspecifics, both in terms of frontal looking at the screen (FL) and contagiousness (YR). Conversely, since caretakers daily spend a considerable amount of time with animals, their faces and yawns are probably processed as those of ‘ingroup’ companions. Stimuli produced by extensively known humans might be easier to be processed and thus might be more contagious than those emitted by stranger faces. Our results also highlight the ability of mangabeys to discriminate between familiar and unfamiliar human faces. Although the capacity to process universal face prototypes is thought to be present at birth, it is highly adaptive that face recognition abilities narrow in the course of ontogeny and reach a high specificity later in life^53^. This can lead to a scarce competence in discriminating familiar vs unfamiliar heterospecific faces. Yet, early experience can maintain a certain degree of plasticity in the primate face recognition system^54^. The daily experience that our mangabeys have with caretakers are probably at the basis of their ability to properly process human faces.

Recent data suggests that the propensity to mimic ingroup subjects has been favored by natural selection to increase behavioral synchronization, fundamental for survivorship and improvement of individual fitness^55^. For example, YC in lions translates into a higher probability to align social activities that are at the basis of cooperative hunting, offspring care, and territorial defense^6^. Other forms of behavioral synchronization have been reported to be extremely adaptive (vigilance against predators^56^). It is possible that the natural propensity to be behaviorally infected by group mates could expand to familiar subjects of different species under contexts allowing interspecies interactions (e.g., captivity). Experimental data demonstrate that capuchin monkeys preferentially engaged in objects sharing with experimenters who previously imitated them compared to experimenters who performed non-imitative gestures^57^. This finding is in line with our results on the importance of familiarity in modulating YC despite the inter-specific context. Here, the adaptive value might reside in the possible linkage between motor resonance phenomena (YC) and the resource benefits (e.g., food provisioning) gathered by monkeys from their caretakers.

In conclusion, in mangabeys YC is socially modulated not only at intra- but also at interspecific level, suggesting that the phylogenetic closeness of the interacting subjects is not enough to explain the phenomenon. Other factors such as the social feature of a species (e.g., social system, preferential relationships, ingroup competition) and the ontogenetic pathways of each individual (e.g., rearing conditions, more or less opportunities of early experiences, degree of plasticity in forming bonds) should be taken into account to fully explain the propensity to YC and its modulation from an adaptive perspective.

## Supplementary Information

Supplementary Information 1.

Supplementary Information 2.

Supplementary Information 3.

## Data Availability

The raw data supporting the conclusions of this article are provided as supporting information to the article.
