# Supplementary material for: Familiarity modulates both intra- and interspecific yawn contagion in red-capped mangabeys
Source: Sci Rep. 2022 Jul 1;12:11138. doi: 10.1038/s41598-022-15395-0 (PMC9249767; doi:10.1038/s41598-022-15395-0)
Supplement: Supplementary file 1 — Supplementary Information 1. [file 41598_2022_15395_MOESM1_ESM.pdf]

FAMILIARITY MODULATES BOTH INTRA- AND INTERSPECIFIC YAWN CONTAGION IN RED-CAPPED MANGABEYS

Luca Pedruzzi, Juliette Aychet, Lise Le Vern, Veronica Maglieri, Arnaud Rossard, Alban Lemasson, Elisabetta Palagi

SUPPLEMENTARY INFORMATION

EXPERIMENTAL PROCEDURE

The red-capped mangabeys part of the study (10 males and 7 females) were captivity-born between 1992 and 2015 and are hosted at the Station Biologique de Paimpont (University of Rennes 1). The animals occupy enclosures with indoor and outdoor spaces (from 8 to 26.4 m² for indoor, 14.7 to 37.2 m² for outdoor enclosures; height from 2.5 m to 4.4 m). indoor enclosure the temperature was kept at 22 °C. Water was available ad libitum and the animals were provided with two meals per day (12.30 - 4.30 p.m., one with fresh fruits and vegetables, the other one with monkey chow). The experiments were not carried out in case of rain or outside temperature below 6 °C.

In the experimental sessions the screen (Sharp LC-46XL2E TV 46") was positioned to maximize the probability of image detection from the tested subject, and to minimize that for the outside non-tested subjects, to prevent them from getting habituated or losing interest in the screen. Two cameras (Sony HDR-XR155E and JVC Full HD GZ-RX615) were located to cover all the test area. As soon as the subject spontaneously entered the testing area, the experimenter switched on the screen and cameras and immediately left the room. The experiments were performed far from the feeding time and each subject was not tested more than once per day.

VIDEO ANALYSIS AND STATISTICS

BORIS v.7.9.19 software (1) was used by L.P. to analyze the videos of the sessions. Concerning the variables coded, to avoid the risk of autocorrelation (a yawn can elicit another yawn in the same subject), the YR variable (binomial variable) was defined as the presence/absence of yawn response in the 8 minutes of videos (5-min stimulus and 3-min post-stimulus). The FL variable is a rate obtained as the seconds spent in FL on the 5 minutes of stimulus duration (5-min stimulus). The SDB variable is a rate obtained as the seconds spent in SDB on the 8 minutes of videos (5-min stimulus and 3-min post-stimulus). To apply Gaussian distribution models (LMM), FL and SDBs were respectively square-root and log-transformed.

Inter-observer reliability was tested on about 20% of total videos, for yawns (presence, exact time) and self-directed behaviors (presence, exact time). During the video analysis, L.P. was not aware of the stimulus provided to the subjects (the stimulus depicted on the screen was not even visible in the videos of the sessions). V.M. also checked the videos without knowing which stimulus the tested subject was perceiving (blind condition). L.P. and V.M. obtained a Cohen k coefficient of 0.93 for yawning, 0.87 for FL and 0.99 for the SDBs.

For both Model set_1 and Model set_2, we first included meaningful interactions between the predictors: *Sex*Condition, Sex*Species* (Model set_1) and *Sex*Condition, Sex*Species, Familiarity*Species, Familiarity*Sex* (Model set_2). To reach reliable results a bare minimum of 10 observations per predictor is suggested (2), and the size of our sample (i.e., 102 sessions Model set_1 and 136 Model set_2) may lead to non-precise estimations when introducing too many fixed effects (e.g., interaction terms) (3). For these reasons, the interactions were included in each final model only if they were significant.

We verified the normal distribution and homogeneity of the model's residuals by looking at the Q-Q plot and plotting the residuals against the fitted values (4). Multicollinearity in the GLMMs was checked with the 'check_collinearity' function from the R package *performance*

0.4.4 (by means of VIFs): 'Low correlation' was found for all the parameters in the six GLMMs (VIF range: 1.00-1.41). The significance of the models was tested comparing the full model with the model including only the random effects (i.e., null model) (5) through the Likelihood Ratio Test (LRT, Anova with the 'Chisq' test argument, 6). To estimate each predictor p-value, LRTs were run between the full model and the model not containing that predictor (using the 'drop1' function; 7). To check the models fit and possible overdispersion issues the package DHARMa 0.3.3.0 (8) was used (for the six models, dispersion range: 1.007-1.032, p-value range: 0.75-0.88). The R-package MuMIn 1.43.17 (9) was used to calculate the marginal and residual R^2; the marginal R^2 indicates the proportion of variance of the response variable explained by the fixed factors only, whereas the residual R^2 indicates that one explained by both fixed and random factors (10). Relative odds ratios were used to show the actual influence of the estimated effects (in the models with binomial distribution), using the 'confint()' function; OR indicate the expected odds change, when all the variables are kept at the reference value, when the fixed factor increases by one unit. The pairwise comparisons for the factor with more than two levels (Species) in the $Model_{1a}$ were done using the R package emmeans performing the Tukey test (11,12). All analyses were performed with RStudio 1.4.1106 (The R Foundation for Statistical Computing, Vienna, Austria, http://www.r-project.org).

1. Friard, O., Gamba, M. BORIS: a free, versatile open-source event-logging software for video/audio coding and live observations (2016) https://doi.org/10.1111/2041-210X.12584
2. Bolker, B. M. *et al*. Generalized linear mixed models: a practical guide for ecology and evolution. *Trends ecol evol*, *24*(3), 127-135 (2009)

3. Kumle, L., Võ, M. L. H., Draschkow, D. Estimating power in (generalized) linear mixed models: An open introduction and tutorial in R. *Behav Res Methods*, 53(6), 2528-2543 (2021)
4. Estienne, V., Mundry, R., Kühl, H. S., Boesch, C. Exploitation of underground bee nests by three sympatric consumers in Loango National Park, Gabon. *Biotropica*, 49(1) 101-109 (2017)
5. Forstmeier,W., Schielzeth, H. Cryptic multiple hypotheses testing in linear models: Overestimated effect sizes and the winner's curse. *Behav Ecol Sociobiol*, *65*(1), 47–55 (2011)
6. Dobson, A. J., Barnett, A. G. An introduction to generalized linear models (2nd ed.). Boca Raton, FL: Chapman and Hall/CRC press. (2018)
7. Barr, D. J., Levy, R., Scheepers, C., Tily, H. J. *Random effects structure for confirmatory hypothesis testing: Keep it maximal* (2013) https://doi.org/10.1016/j.jml.2012.11.001
8. Hartig, F. DHARMa: Residual Diagnostics for Hierarchical (Multi-Level/Mixed) Regression Models. version 0.3.3.0. R package (2020) https://CRAN.R-project.org/ package=DHARMa.
9. Bartoń, K. MuMIn: Multi-Model Inference. R package version 1.43.17 (2020) https:// CRAN.R- project.org/ package= MuMIn . Accessed 06 Aug 2021.
10. Nakagawa, S., Johnson, P. C. D., Schielzeth, H. *Appendix S6: Worked examples for "The coefficient of determination R 2 and intra-class correlation ICC from generalized linear-mixed effects models revisited and expanded."* (2017)
11. Bretz, F., Hothorn, T., Westfall, P. Multiple Comparisons Using R (1st ed.). *Chapman and Hall/CRC* (2010) https://doi.org/10.1201/9781420010909
12. Lenth, R. *et al.* Package "emmeans". https://cran.r-project.org/web/packages/emmeans/emmeans.pdf (2021) Accessed 06 Aug 2021

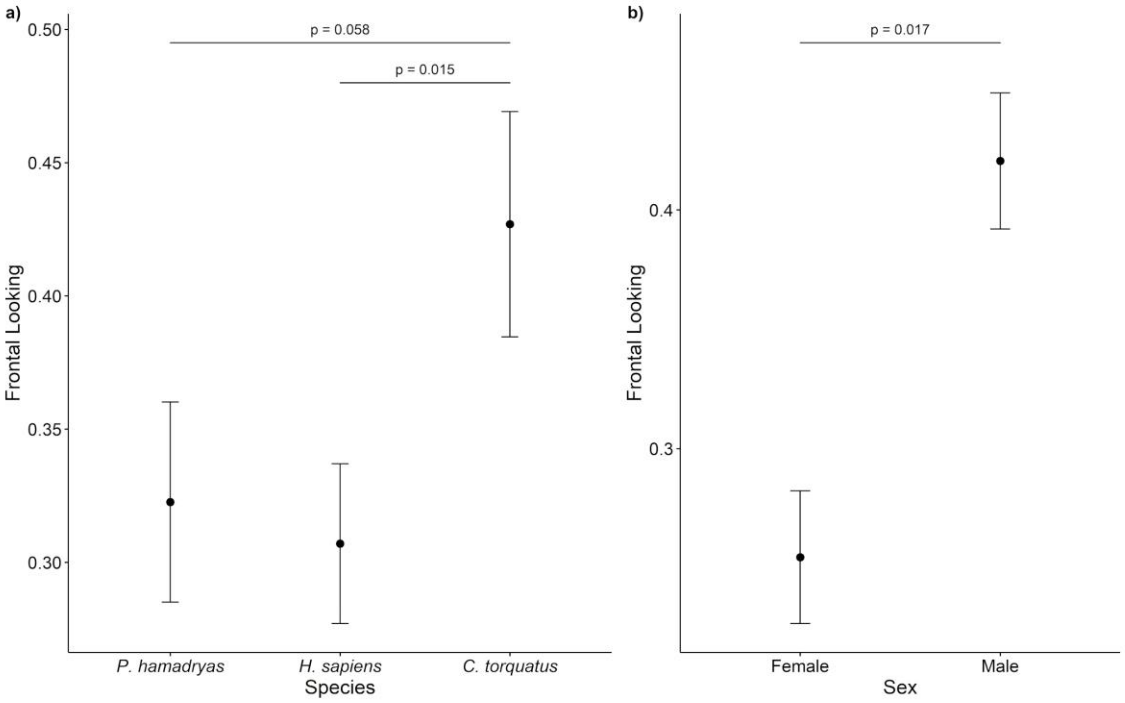

Supplementary Figure 1 - a) Mean rate of Frontal looking ± SE when videos depicted hamadryas baboons, humans, or mangabeys. P-values indicate the results of Tukey tests (*C. torquatus* vs *H. sapiens*: t-ratio= -2.847; df = 93; p = 0.015. *C. torquatus* vs *P. hamadryas*: t-ratio = -2.320; df = 93; p = 0.058. *H. sapiens* vs *P. hamadryas*: t-ratio = 0.506, df = 93, p = 0.869). **b)** Mean rate of Frontal looking ± SE when the tested subject was male *vs* female ($Model_{1a}$, n = 102, Sex: $\chi 2 = 5.692$, P = 0.017; full results: Table 1a).

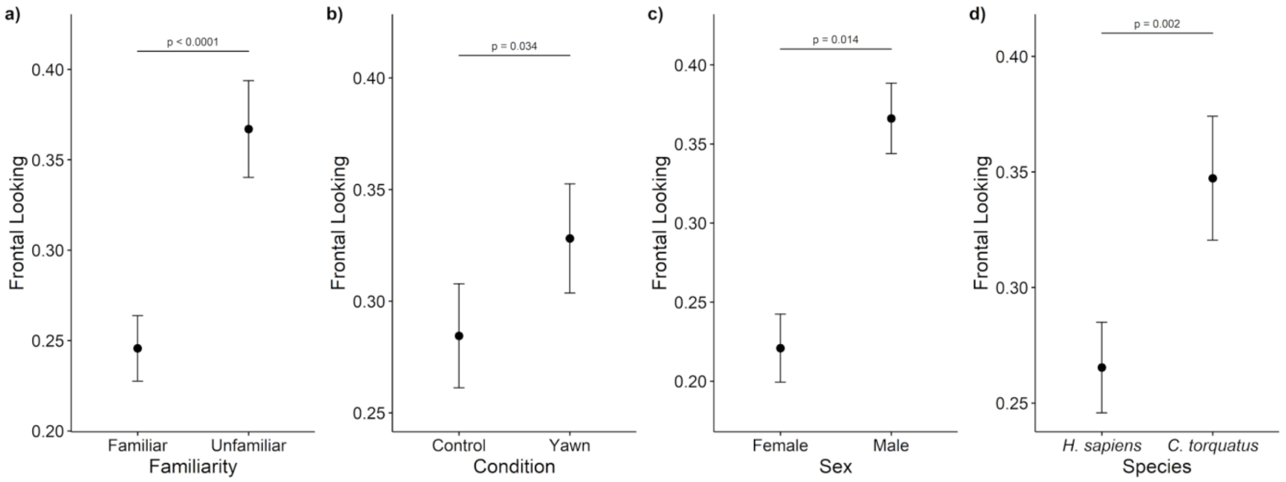

Supplementary Figure 2 - a) Mean rate of Frontal Looking ± SE when videos depicted familiar *vs* unfamiliar subjects ($Model_{2a}$, n = 136, Familiarity: $\chi2 = 21.012$, $P < 0.0001$; full results: Table 2a). **b)** Mean rate of Frontal Looking ± SE when the videos depicted humans *vs* mangabeys ($Model_{2a}$, n = 136, Species: $\chi2 = 10.073$, $P = 0.002$; full results: Table 2a). **c)** Mean rate of Frontal Looking ± SE when the videos were in the Yawn *vs* Control condition ($Model_{2a}$, n = 136, Condition: $\chi2 = 4.518$, $P = 0.034$; full results: Table 2a). **d)** Mean rate of Frontal Looking ± SE when the tested subject was a male *vs* female ($Model_{2a}$, n = 136, Sex: $\chi2 = 6.056$, $P = 0.014$; full results: Table 2a).
